# Editorial: Advances and challenges of genome engineering tools in healthcare: molecular insights into CRISPR/Cas technology

**DOI:** 10.3389/fmolb.2024.1376509

**Published:** 2024-03-11

**Authors:** Sanjay Kumar, Sumana Venkat

**Affiliations:** ^1^ Biological and Bio-computational Lab, Department of Life Science, Sharda School of Basic Sciences and Research, Sharda University, Greater Noida, India; ^2^ University of Texas Southwestern Medical Center, Dallas, TX, United States

**Keywords:** gene editing, CRiSPR/Cas, overcoming drug resistance, therapy, healthcare

Currently, a diverse array of treatment options is available for various diseases, encompassing surgery, radiation, hormone therapy, chemotherapy, and targeted therapeutics. However, it is crucial to note that these therapeutic approaches often entail a range of adverse reactions. The ongoing pursuit of innovative treatments with minimal negative side effects for patients is a constant priority. In line with this perspective, genome therapy emerges as an enticing approach for monitoring mutations and treating various diseases, including cancers. Established gene-editing tools such as zinc-finger nucleases (ZFNs) and transcription activator-like effector nucleases (TALENs) have proven to be efficient. Nevertheless, the recent advent of clustered regularly interspaced short palindromic repeats (CRISPRs) has revolutionized gene editing, transcending both ZFNs and TALENs across numerous therapeutic domains, from laboratory research to clinical applications. The simplicity and precision of CRISPR-Cas-driven gene editing, facilitated by the Watson–Crick base-pairing process between single-guide RNA (sgRNA) and target DNA, render it a more versatile and efficient method. Additionally, the swift and accurate techniques employed in this approach contribute to its cost-saving advantages. Notably, the risk-minimized strategy ensures that off-target impacts are minimized (Karn et al., 2022). In this context, our Research Topic was launched in 2022, inviting scientists to contribute their research on molecular insights into CRISPR/Cas technology in healthcare. The aim is to advance our understanding and application of this technology to save lives at both individual and societal levels.

The CRISPR-Cas system is made up of two components, namely, CRISPR and CRISPR-associated (Cas) proteins. CRISPR with dyad symmetry structure was first identified in *Escherichia coli*, which has been associated with immune function in the bacterial system (Ishino et al., 1987). The mechanisms of the CRISPR/Cas system consist of three different steps: 1) adaptation, where Cas proteins recognize and identify foreign DNA that get fragmented and incorporated into the CRISPR array; 2) expression, in which the CRISPR array becomes transcribed into mature CRISPR RNAs (crRNAs); and 3) interference, in which the spacer sequence of the crRNA captures foreign DNA, which is further cut by Cas proteins, with nuclease activity (Alkhnbashi et al., 2020). However, these various Cas proteins have been associated with numerous functions in these three steps of the CRISPR/Cas mechanism. These Cas proteins may be classified into core and auxiliary Cas proteins. Furthermore, core Cas proteins are majorly involved in the immune process of CRISPR/Cas, whereas auxiliary Cas proteins have been associated with other steps of CRISPR/Cas involved in immunity. Currently, there have been 13 different types of Cas core proteins identified, namely, Cas1, Cas2, Cas3, Cas4, Cas5, Cas6, Cas7, Cas8 (large subunit), Cas9, Cas10 (large subunit), Cas11 (small subunit), Cas12, and Cas13 (Yang et al., 2021). Cas2 is known for the function of adaptive immunity against foreign nucleic acids. However, the subtype I-C of Cas does not contain the CRISPR element required for RNA-driven adaptive immune function against exogenous nucleic acids. The logic to sustain the function of Cas genes in the absence of a CRISPR array was not known. To address this, Anand et al., published the article “Structural and functional characterization of Cas2 of CRISPR-Cas subtype I-C lacking the CRISPR component” as part of this Research Topic, where it was shown that the recombinant Cas2C expressed metal-dependent DNase and metal-independent RNase activities (Anand et al.), suggesting that in the absence of the CRISPR array, Cas2C is very crucial in processes, which is distinct from the CRISPR-Cas-associated function.

The labeling of the cell membrane is very important to study the membrane structural insight of bacteria. In this context, Nickels et al. published an article titled “Improved chemical and isotopic labeling of biomembranes in *Bacillus subtilis* by leveraging CRISPRi inhibition of beta-ketoacyl-ACP synthase (fabF)” as a part of this Research Topic, where they described that blocking of the transcription of the essential fabF gene utilizing dCas9/sgRNA-fabF in the presence of exogenous xylose resulted in enhanced sensitivity to antifungal cerulenin, an inhibitor of fatty acid (FA) synthesis in the mutant strain (JEBS102) of *B. subtilis.* Furthermore, it also boosted cell growth via increased uptake of FA in the presence of exogenous FA in the culture medium. This demonstrated that utilizing the CRISPR tool led to an improved membrane labeling system as compared to other previously reported methods such as small-angle neutron scattering, which used genetic inhibition of FA degradation (Δ*fadN*).

The understanding of the working mechanism of CRISPR/Cas9 to editing a specific gene is very crucial. Cas9 is a versatile molecule that undergoes various functional states. To comprehensively grasp the distinct functions of each state of Cas9, a holistic approach is essential, involving the integration of computational and experimental methods. In the article “Twisting and swiveling domain motions in Cas9 to recognize target DNA duplexes, make double-strand breaks, and release cleaved duplexes” published as a part of this Research Topic, Wang et al. reviewed various biochemical, structural, and biophysical studies of mechanisms that show how Cas9 is involved in identifying the target DNA duplex and denies non-specific target sequences for specific gene editing.

Efficient, targeted, and reliable diagnostic tools for the prompt identification of viruses and pathogens are essential to effectively manage the global spread of diseases. Among the various diagnostic approaches for detecting COVID-19 infection, CRISPR-based nucleic acid tests stand out as a leading and notable method. Aouida et al. published the article “A CRISPR-based approach using dead Cas9-sgRNA to detect SARS-CoV-2” in this Research Topic. They developed a novel CRISPR/Cas9-based specific and rapid diagnostics method to detect the M-gene of SARS-CoV-2 utilizing an *in vitro* dCas9/sgRNA system (Aouida et al.). Moreover, utilizing the dead Cas9 variant to safeguard restriction enzyme sites is a noteworthy aspect of this method. This technique holds promise for being employed as a diagnostic tool for a wide range of DNA and RNA pathogens.

Several challenges and limitations have been associated with CRISPR/Cas systems, which are very important to address for its successful therapeutic utility in specific gene editing. Keeping this in view, Tiwari et al. published the article “CRISPR/Cas9 as a therapeutic tool for triple negative breast cancer: from bench to clinics” in this Research Topic. They comprehensively reviewed the new development, challenges, limitations, and aspects of CRISPR/Cas9 for the management of triple-negative breast cancer (TNBC). The poor delivery system, off-target effects, and social and ethical concerns due to genetic changes were noted to be major challenges associated with CRISPR. Additionally, the authors also highlighted that integrating artificial intelligence and machine learning could be very important in advancing CRISPR/Cas9-based therapeutic approaches for TNBC management.

Conventional approaches to investigating miRNA (microRNAs) functions, such as miRNA inhibitors and sponges, have many drawbacks in terms of specificity, short-lived effects, and unintended off-target impacts. Similarly, the application of CRISPR/Cas9-based editing on miRNAs through sgRNAs faces limitations in terms of the design and flexibility required for generating highly efficient gRNAs. Keeping this in view, Ijee et al. published the article titled “Efficient Deletion of MicroRNAs Using CRISPR/Cas9 with Dual Guide RNAs” as a part of this Research Topic, where they developed a novel dual guide RNA (dgRNA) along with CRISPR/Cas9 for the fast and efficient deletion of miRNAs. Hence, dgRNA-driven miRNA deletion could be utilized to make efficient pooled libraries for the comprehensive study of miRNAs associated with various physiological functions.

Moreover, CRISPR has been widely used in clinical trials to change somatic cells *ex vivo* with the goal of lowering risks and then transferring into *in vivo* gene therapeutic purposes (Charlesworth et al., 2019). Nevertheless, germline gene editing research for therapy continues to confront ethical obstacles. In this sense, present and upcoming clinical trials on somatic CRISPR therapy must be reviewed over time to ensure system efficacy and safety.

Despite encountering technical obstacles in targeting oncogenes, the prospects for gene therapy utilizing CRISPR/Cas9 remain promising. In the future, tailored treatments employing CRISPR/Cas9-based approaches could be a successful strategy for addressing the complexities associated with a variety of tumors and resistance to cancer treatments. However, the efficacy of CRISPR/Cas9-driven therapy hinges on the quality of sgRNA design, thorough monitoring for off-target effects, and the development of effective delivery methods. This technique has displayed significant potential in addressing chemotherapy drug resistance across all stages of investigation.
